# Topical recombinant thrombin at a concentration of 1000 IU/mL reliably shortens in vivo TTH and delivers durable hemostasis in the presence of heparin anticoagulation and clopidogrel platelet inhibition in a rabbit model of vascular bleeding

**DOI:** 10.1186/1750-1164-3-14

**Published:** 2009-11-19

**Authors:** Steven D Hughes, Paul D Bishop, Richard Garcia, Tracy Zhang, W Allan Alexander

**Affiliations:** 1 ZymoGenetics, Inc Seattle, WA, USA

## Abstract

**Background:**

This study was designed to evaluate the effect of recombinant human thrombin (rThrombin) concentration on time to hemostasis (TTH), clot durability, and clot strength in settings that replicate the heparinization and platelet inhibition often found in surgical populations.

**Methods:**

A modified, anticoagulated rabbit arteriovenous shunt preparation was selected to model vascular anastomotic bleeding. Rabbits were treated with heparin or heparin + clopidogrel and TTH was measured after applying a range of topical rThrombin concentrations or placebo, in combination with absorbable gelatin sponge, USP. Treatments (placebo, rThrombin) were randomly assigned and the investigator was blinded to treatment. TTH was evaluated with the Kaplan-Meier method. After hemostasis was achieved, clot burst assessment was performed for heparin + clopidogrel treated animals. Clot viscoelastic strength and kinetics were measured in ex-vivo samples using thromboelastography (TEG) methods.

**Results:**

TTH decreased with increasing concentrations of rThrombin in heparin-treated animals and was shorter after treatment with 1000 IU/mL rThrombin (73 seconds) than with 125 IU/mL rThrombin (78 seconds; p = 0.007). TTH also decreased with increasing concentrations of rThrombin in heparin + clopidogrel treated animals; again it was significantly shorter after treatment with 1000 IU/mL rThrombin (71 seconds) than with 125 IU/mL rThrombin (177 seconds; p < 0.001). Variability in TTH was significantly smaller after treatment with 1000 IU/mL rThrombin than after 125 IU/mL rThrombin, indicating greater reliability of clot formation (p < 0.001 for heparin or heparin + clopidogrel treatments). Clot durability was examined in heparin + clopidogrel treated animals. Clots formed in the presence of 1000 IU/mL rThrombin were significantly less likely to rupture during clot burst assessment than those formed in the presence of 125 IU/mL rThrombin (0% versus 79%, p < 0.001). In vitro clot strength and clot kinetics, as determined by TEG in heparin + clopidogrel samples, were positively associated with the amount of rThrombin activity added for clot initiation.

**Conclusion:**

In an animal model designed to replicate the anti-coagulation regimens encountered in clinical settings, topical rThrombin at 1000 IU/mL more reliably controlled the pharmacological effects of heparin or heparin + clopidogrel on hemostasis than rThrombin at 125 IU/mL. Results from in vitro assessments confirmed a positive relationship between the amount of rThrombin activity and both the rate of clot formation and clot strength.

## Background

In the study described herein, a rabbit model of arterial anastomotic bleeding was used to examine the effect of recombinant human thrombin (rThrombin) concentration on time to hemostasis (TTH) under varying conditions of pharmacologic anticoagulation and platelet inhibition.

Thrombin has been widely used as a topical hemostatic agent during surgical procedures since the 1940s [1,2]. The efficacy of topical thrombin at a concentration of 1000 IU/mL was recently demonstrated in clinical trials, although no clinical trials have compared the effects of different concentrations of topical thrombin on hemostatic efficacy [3-5]. The critical role of thrombin concentration in fibrin clot formation has been demonstrated in a number of in vitro settings; clots formed in the presence of higher concentrations of thrombin had more tightly packed fibrin strands and were more resistant to fibrinolysis, when compared with clots formed in the presence of lower concentrations of thrombin [6-8]. In animal models, TTH was dependent on rThrombin concentration; for example, gauze soaked in rThrombin at concentrations from 500 to 2000 IU/mL stopped bleeding significantly faster in a rabbit model of hepatic bleeding than gauze soaked with rThrombin 100 IU/mL [9,10]. In another study, 125 IU/mL human plasma-derived thrombin did not appear to be an adequate concentration for establishing durable clots in a healthy porcine model of liver injury [11]. In that study, human plasma-derived thrombin (125 IU/mL) with an absorbable gelatin sponge (AGS) improved the incidence of hemostasis compared to saline with AGS. However, rebleeding was also observed at a number of sites during the 12 minute evaluation period, thus thrombin at 125 IU/mL did not appear to be an adequate concentration for establishing durable clots. Although there is substantial inter-species heterogeneity in coagulation capacity, collectively these results suggest that there is an optimum concentration of topical thrombin needed at the wound for rapid TTH and durable clotting.

Clopidogrel is a potent platelet function inhibitor and is widely prescribed, ranking second in a 2008 list of worldwide pharmaceutical sales [12]. There are a wide variety of opinions regarding the timing for discontinuing clopidogrel before undergoing a surgical procedure. After recent reports of rebound cardiac risk for 90 days following abrupt discontinuation of clopidogrel, it has been argued that stopping clopidogrel in patients with a high risk of perioperative myocardial infarction may affect the incidence of perioperative cardiac events [13]. As a consequence, increasing numbers of patients are presenting for emergent, urgent, or elective surgical procedures with significant clopidogrel platelet inhibition as a result of a reluctance or inability to withhold clopidogrel prior to surgery. The effect of anti-platelet medications on TTH has not been thoroughly evaluated, although recent clinical trial data suggested that TTH was delayed in patients being treated for conditions for which anti-platelet medications are commonly-used when compared with patients who were receiving treatment for conditions for which anti-platelet medications are less-commonly used [14].

Based on the observations described above, we hypothesized that adoption of the standard 1000 IU/mL thrombin concentration gained acceptance in clinical practice over the last 50 years because of its observed efficacy over a range of clinical settings. Although thrombin is a remarkably potent effector of hemostasis, the conditions in which exogenous thrombin is applied vary widely, with differences in blood flow, the amount of hemodilution, and the presence or absence of pharmacological anticoagulation agents. Considering these variables, one would predict that topical thrombin applied at lower concentrations may be ineffective in some clinical settings.

In this study, the effect of rThrombin concentration on TTH under varying conditions of pharmacologic anticoagulation and platelet inhibition was evaluated by performing in vivo experiments with a range of thrombin concentrations in a model of arterial anastomotic bleeding, in rabbits treated with heparin or with heparin + clopidogrel. Reliability of clot formation was assessed by examining variability in TTH. After hemostasis was achieved, we examined differences in clot durability resulting from various concentrations of rThrombin by evaluating clot burst in animals treated with heparin + clopidogrel. We also performed an in vitro assessment of clot strength and the rate of clot formation by adding different amounts of rThrombin activity to heparin + clopidogrel rabbit blood samples, and evaluating the viscoelastic properties of the clot using modified thromboelastography (TEG) [15].

## Methods

### Animals

Female New Zealand White rabbits (Charles River Laboratories, Hollister, CA), weighing 2.0 to 3.8 kg and approximately 12 weeks of age, were used for these studies. Six animals were used in each study. Animals were acclimated to the facility for 7 to 10 days before the surgical procedure and were maintained in good condition. All animal studies were performed under approved protocols in accordance with the Guide for the Care and Use of Laboratory Animals (US DHHS publication NIH 86-23).

### In Vivo Study Design

Clinical vascular anastomotic bleeding was simulated in a rabbit arteriovenous (AV) shunt model. The experimental design evaluated the dependence of TTH on the applied rThrombin concentration in the presence of heparinization, both with and without clopidogrel platelet inhibition. All rThrombin concentrations were applied using an AGS. Anticoagulation with heparin was used in this model in order to maintain shunt patency. An initial study, evaluating several rThrombin concentrations (31.25, 62.5, 125, and 1000 IU/mL) or placebo, was performed in rabbits treated with heparin. In further studies, hemostasis was evaluated in rabbits treated with heparin + clopidogrel using two concentrations of rThrombin (125 and 1000 IU/mL) or placebo. The investigator was blinded to treatment during the procedure and at the time of hemostasis evaluation, and treatment (placebo, rThrombin) was randomly assigned.

Multiple measurements of TTH were performed on each rabbit. Each measurement was performed on a new polytetrafluoroethylene (PTFE) graft. In the initial study of rabbits treated with heparin, 12 grafts were evaluated for placebo and 8 grafts were evaluated for each rThrombin concentration. In the heparin + clopidogrel treated rabbits, 10 grafts were evaluated for placebo and 14 grafts were evaluated for each rThrombin concentration. For each graft, physiological parameters critical to hemostasis (i.e., model control parameters) were measured. These included body weight, activated partial thromboplastin time (aPTT; measured at baseline and after heparin treatment), body temperature, blood flow, and mean arterial pressure (MAP). Comparability of these parameters was used to demonstrate consistency among animals and standardization between grafts, and was necessary in order to consider each measure of TTH in an individual graft as an independent observation.

### Treatments: rThrombin, Placebo, and Absorbable Gelatin Sponge

On the day of the AV graft procedure, a 5000 IU vial of rThrombin (RECOTHROM^®^, ZymoGenetics, Inc., Seattle, WA) was reconstituted in 5 mL of sterile saline, yielding a 1000 IU/mL rThrombin solution. The 1000 IU/mL solution was diluted with placebo to yield 31.25 IU/mL, 62.5 IU/mL, and 125 IU/mL rThrombin solutions. Placebo solution consisted of the excipients contained in the formulation for rThrombin [16]. AGS (Gelfoam, Pharmacia & Upjohn Company, Kalamazoo, MI, size 100) was cut into 2 × 4 × 1 cm strips and combined with rThrombin or placebo solutions in accordance with the instructions in the rThrombin package insert. The median final volume of rThrombin or placebo mixed per sponge was 0.762 mL (range 0.756-0.775 mL).

### Clopidogrel Administration

Animals received 20 mg/kg oral doses of clopidogrel bisulfate (Plavix^®^, Bristol-Meyers Squibb/Sanofi Aventis, Bridgewater, NJ) once daily for three days prior to AV shunt placement and hemostasis evaluation. A 75 mg clopidogrel tablet was dissolved in 3 mL of sterile water. Each animal received between 1.6 and 2.2 mL of the 25 mg/mL solution per day by gavage. Surgical procedures were performed 2 hours after the final dose of clopidogrel.

### Rabbit AV Shunt Procedure

AV shunts were surgically created by using a modification of methods previously described [17]. Briefly, rabbits were immobilized with ketamine hydrochloride (Fort Dodge Animal Health, Ft. Dodge, IA; 50 mg/kg, intramuscular injection), and anesthesia was maintained using inhaled isoflurane (1% to 2%). The animals were placed on a water-jacketed heating pad maintained at 37°C during the experimental period and core body temperature was monitored. The animal's right femoral artery was cannulated for measurement of mean arterial pressure (MAP) and the left femoral vein was cannulated for heparin administration. An AV shunt that linked the blood flow of the left carotid artery and the right jugular vein was created by isolating and cannulating these vessels with a 3 to 4 cm length of Micro-Renathane tubing (MRE 080, 0.080" O.D. × 0.040" I.D., Braintree Scientific, Braintree, MA) connected to a 15 cm length of silicone catheter tubing (7-French, 0.078" I.D. × 0.125" O.D., Access Technologies, Skokie, IL). The catheters were exteriorized and connected with a 2 to 2.5 cm PTFE graft segment (3 mm, SN AFEP 7108, Bard Peripheral Vascular Incorporated, Tempe, AZ). Blood flow through the shunt was measured using a Transonic System Incorporated Flowmeter, model TS410 (Ithaca, NY). Each rabbit received 100 U/kg heparin (Abraxis Pharmaceutical Products, Schaumburg, IL) by intravenous (IV) bolus injection, followed by a continuous 50 U/kg/mL infusion of heparin at a flow rate of 5 mL/hr via the femoral vein; aPTT was measured prior to heparin treatment and 5-10 minutes after IV heparin bolus.

### Suture Hole Bleed and Measurement of TTH

To achieve consistent conditions between AV grafts, MAP was maintained at approximately 55 mm Hg (+/- 2 mm Hg) by adjusting depth of anesthesia, and aPTT was maintained at a value > 400 seconds by heparin infusion. Suture hole bleeding was assessed by puncturing the center section of the PTFE graft segment with two 18 inch silk suture needles (4-0 needles; reverse cutting needle size P-3, stock number 641G, Ethicon Incorporated, Somerville, NJ), creating 4 needle holes. The AGS containing 0.77 mL of rThrombin or placebo was immediately wrapped around the graft, completely covering the bleeding surfaces (suture holes). The wetted AGS was then covered with gauze sponges, and continuous digital pressure was applied. After 60 seconds the gauze sponges were visually inspected for bleeding. If cessation of bleeding had not occurred, new gauze sponges were applied with digital pressure and this process was repeated until no visible blood was observed on the gauze sponges. TTH was recorded in seconds. If TTH was not achieved within 300 seconds, the experiment was terminated. At the conclusion of the assessment period, the catheters were clamped, flushed with saline, the PTFE segment was removed, and a fresh PTFE segment was inserted. Baseline measurements were obtained for MAP, core temperature, and aPTT. Additional heparin (50 U/kg, IV bolus) was injected as needed to maintain an aPTT > 400 seconds. If a MAP of 55 mm Hg could not be maintained by adjusting the depth of anesthesia, the animal was euthanized.

### Suture Hole Clot Burst Assessment

In the rabbits treated with heparin + clopidogrel, clot burst at the suture needle puncture sites was assessed after the 300 second assessment of TTH, if hemostasis had been achieved. The procedure involved clamping off blood flow to the jugular vein catheter approximately 2-3 cm downstream from the AV shunt graft for a period of 10 seconds. Complete obstruction of blood flow created an increase in blood pressure at the site of the blood clot. Clot burst observation during the 10 second period was recorded as either positive (blood seeped through the AGS onto the gauze sponge) or negative (no seepage was observed). Clot burst was not assessed in rabbits treated with heparin alone.

### In Vitro Study Design

The viscoelastic properties of blood clots were assessed in ex-vivo blood samples. TEG was used to measure the mechanical resistance to clot formation as a function of time and provided an in vitro correlate for the effects of rThrombin concentration on the rate of clot formation and clot strength.

### TEG Samples from Rabbit AV Shunt Model

Blood samples from the central ear artery were collected in citrate. For the 3 rabbits evaluated, a sample was collected 2 hours after clopidogrel dosing and heparin (1 U/mL) was added ex vivo. The citrated rabbit blood was recalcified according to a standard protocol with 0.2 M CaCl_2_ plus normal saline just prior to the TEG assay (Haemoscope Corporation, Niles, IL). The blood clotted within normal parameters for rabbit blood without exogenous rThrombin. A modified method for rThrombin clot initiation was empirically derived and covered a ten-fold range of rThrombin enzymatic activity (0.76 to 6.1 IU added per mL of rabbit blood).

### Thromboelastograph Parameters

TEG measures clot viscoelastic properties by recording mechanical resistance to clot formation as a function of time.

• The rate of clot formation (i.e., development of the fibrin matrix, factor XIII cross-linking, platelet incorporation, and platelet aggregation) is obtained by measuring the alpha angle (α) [15]. The α is derived from the slope of the maximum amplitude (MA) of the measured resistance to clot formation vs. time.

• Clot firmness or strength is measured by the MA and is highly influenced by platelet number and function [15]. Clot strength (shear elastic modulus) is represented by G and is calculated from the amplitude. Maximum clot strength is derived as follows:

The relationships between both the rate of clot formation (α) and clot strength (Gmax) and the amount of rThrombin activity added for clot initiation were assessed.

### Statistical Analyses

Statistical analyses are described below for each experiment and were performed using SAS software (version 9.2, SAS Institute Inc., Cary, NC). Statistical significance was defined as p-values < 0.05. The number of grafts per treatment group was not based on formal sample size calculations or statistical power.

#### Comparison of TTH

Each measurement of TTH in an individual graft was considered an independent measurement. That is, the experimental system (rabbit and shunt) was considered uniform except for the presence or absence of clopidogrel. The effect of different rThrombin concentrations on TTH was evaluated with the Kaplan-Meier method. Comparisons of TTH between different concentrations of rThrombin were performed using the log-rank test. TTH times of > 300 seconds were censored.

#### Variability of TTH

The reliability of hemostasis onset observed after treatment with 125 IU/mL or 1000 IU/mL rThrombin was compared for rabbits treated with heparin and for rabbits treated with heparin + clopidogrel by analyzing the variability in TTH. Hypotheses of equal variability in TTH for rThrombin concentrations of 125 IU/mL and 1000 IU/mL were tested using the folded F-test.

#### Incidence of Clot Burst

After hemostasis had been achieved, we examined potential differences in clot durability induced by different concentrations of rThrombin by evaluating clot burst in rabbits treated with heparin + clopidogrel. Fisher's exact test was used to compare the difference in incidence of clot burst after treatment with 125 IU/mL or 1000 IU/mL rThrombin.

#### In Vitro TEG Assays

Raw TEG data are presented. Pearson correlation coefficients were calculated to assess the relationship between the amount of rThrombin added and the rate of clot formation and clot strength.

## Results

### In Vivo Experiments

A strong concentration-dependent effect of rThrombin on TTH was observed in an initial study in rabbits treated with heparin. Kaplan-Meier estimates of median TTH values were 73 seconds (1000 IU/mL rThrombin), 78 seconds (125 IU/mL), 133 seconds (62.5 IU/mL), 214 seconds (31.25 IU/mL), and > 265 seconds (placebo). Median TTH for the placebo group could not be determined because hemostasis was not achieved before the end of the observation period for half of the 12 grafts (i.e., half the TTH values were > 300 seconds). TTH decreased significantly with increasing rThrombin concentration (p < 0.001). A pairwise log-rank test revealed that TTH after treatment with 1000 IU/mL rThrombin (median 73 seconds; 95% CI: 65, 75) was shorter than after treatment with 125 IU/mL rThrombin (median 78 seconds; 95% CI: 70, 95; p = 0.007). In addition, the reliability of hemostasis onset achieved in grafts treated with 1000 IU/mL rThrombin was greater than that observed in grafts treated with 125 IU/mL rThrombin, as indicated by reduced variability in TTH at the 1000 IU/mL rThrombin concentration in heparin treated animals (p < 0.001).

**Figure 1:**
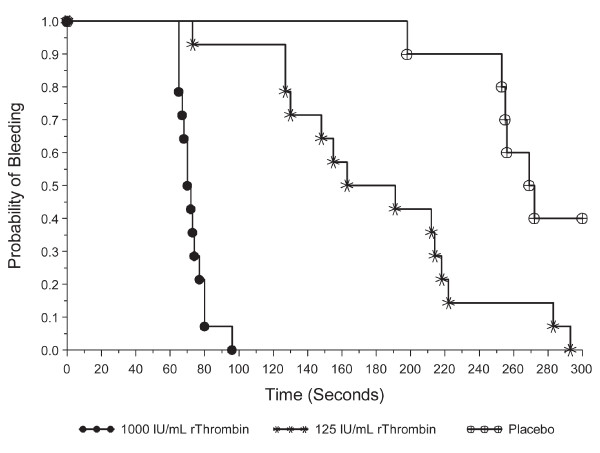
**Kaplan-Meier estimates for the probability of bleeding after treatment with different concentrations of rThrombin or placebo**. TTH was measured in the rabbit AV shunt model of anastomotic bleeding after treatment with heparin + clopidogrel. PTFE graft segments were punctured, and AGS containing a standardized volume of 1000 IU/mL or 125 IU/mL rThrombin or placebo was applied to the bleeding surface. The TTH observation period extended from 60 seconds to 300 seconds. 1000 IU/mL rThrombin: N = 14 grafts. 125 IU/mL rThrombin: N = 14 grafts. Placebo: N = 10 grafts.

A concentration dependent effect of rThrombin on TTH was also observed in subsequent studies in rabbits treated with heparin + clopidogrel (Figure 1). Kaplan-Meier estimates of median TTH were 71 seconds (1000 IU/mL rThrombin), 177 seconds (125 IU/mL), and 271 seconds (placebo). TTH decreased significantly with increasing rThrombin concentration (p < 0.001). A pairwise log-rank test revealed that TTH was significantly shorter after treatment with 1000 IU/mL (median 71 seconds; 95% CI: 65, 77) than after treatment with 125 IU/mL rThrombin (median 177 seconds; 95% CI: 127, 218; p <  0.001).

Looking specifically at the response to 1000 IU/mL rThrombin, median TTH was similar for both treatment regimens (heparin: 73 seconds; heparin + clopidogrel: 71 seconds; p = 0.52). In contrast, median TTH in grafts treated with 125 IU/mL rThrombin was prolonged after heparin + clopidogrel treatment (177 seconds) compared with heparin treatment alone (78 seconds; p < 0.001).

**Figure 2:**
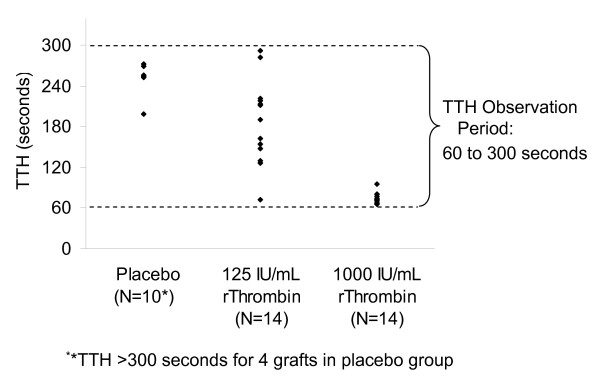
**Variability in time to hemostasis (TTH) after treatment with different concentrations of rThrombin or placebo**. TTH was measured in the rabbit AV shunt model of anastomotic bleeding after treatment with heparin + clopidogrel. PTFE graft segments were punctured, and AGS containing 1000 IU/mL or 125 IU/mL of rThrombin or placebo was applied to the bleeding site. The TTH observation period extended from 60 seconds to 300 seconds, as indicated by the dashed lines. Each diamond represents the TTH value for a single graft. Four grafts in the placebo group had not achieved hemostasis at the end of the observation period and are not included in the graph (i.e. 4 TTH values were > 300 seconds). All grafts treated with 125 IU/mL or 1000 IU/mL rThrombin achieved hemostasis within the observation period.

The reliability of hemostasis onset achieved in grafts treated with 1000 IU/mL rThrombin was greater than that observed in grafts treated with 125 IU/mL rThrombin, as indicated by reduced variability in TTH at the 1000 IU/mL rThrombin concentration in heparin + clopidogrel treated animals (p < 0.001; Figure 2). Variability in the placebo group could not be estimated because hemostasis was not achieved before the end of the observation period for all of the grafts (i.e., some TTH values in the placebo group were > 300 seconds).

**Figure 3:**
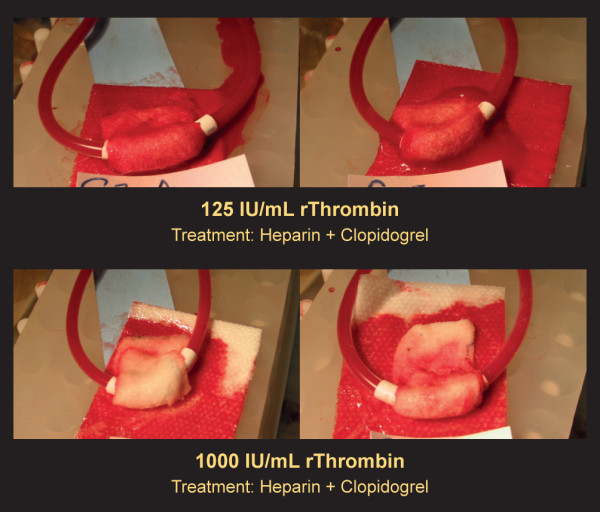
**Clot burst assessment of AV shunt grafts treated with different concentrations of rThrombin**. Rabbits were treated with heparin + clopidogrel. The grafts were wrapped with AGS containing either 125 IU/mL rThrombin (top row) or 1000 IU/mL rThrombin (bottom row). After hemostasis was achieved, blood flow was clamped for 10 seconds, and clot burst was assessed. Images were obtained after the 10 second assessment period. Upper images show sponge saturation and bleed-thru in representative grafts whose hemostasis did not survive clot burst challenge (125 IU/mL rThrombin; 11 of 14 grafts ruptured [79%]). Lower images show representative grafts that maintained hemostasis following clot burst challenge (1000 IU/mL rThrombin; 0 of 14 grafts ruptured [0%]). In the placebo group, most grafts did not maintain hemostasis (8 of 10 grafts ruptured [80%]; data not shown).

Clot durability was assessed in animals treated with heparin + clopidogrel. In these studies, multiple consecutive AV shunt grafts were placed and different topical rThrombin or placebo treatments were administered in each animal. The durability of clots formed in grafts treated with 1000 IU/mL rThrombin was greater than that observed in grafts treated with 125 IU/mL rThrombin, as indicated by reduced incidence of clot burst. Clots that formed in the presence of 1000 IU/mL rThrombin were significantly less likely to rupture during clot burst assessment (0% of clots ruptured) than those formed in the presence of 125 IU/mL rThrombin (79% ruptured; p < 0.001). The incidence of clot burst was comparable in the presence of 125 IU/mL rThrombin (79% of clots ruptured) or placebo (80% ruptured). Representative images of clots that ruptured (upper panel, 125 IU/mL rThrombin) and clots that maintained hemostasis (lower panel, 1000 IU/mL rThrombin) are presented in Figure 3. Ruptured clots (top panel) have blood on the gauze sponges and under the grafts that is not present when hemostasis is maintained (bottom panel).

Data for body temperature, MAP, and blood flow were collected for each graft. Statistical analysis of these data showed that these parameters were consistently maintained (data not shown). Individual body weights and aPTT values at the beginning and end of the study were similar when compared across animals and treatment groups. Terminal aPTT values in each animal were > 400 seconds. This represented a 2.6-fold or greater increase in aPTT from baseline values, an expected effect of the heparin dosing regimen.

### In Vitro TEG Assays

**Figure 4:**
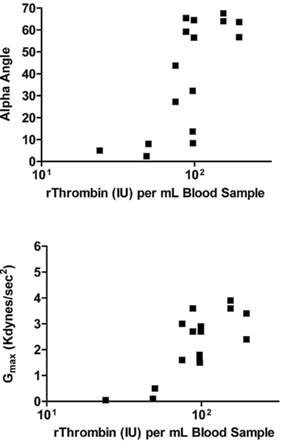
**Effect of rThrombin concentration on rabbit blood thromboelastograph (TEG) parameters**. Blood samples were collected from study animals (N = 3) two hours after they had been treated with clopidogrel, and heparin was added to 1 U/mL. The top panel shows the alpha angle, which is a function of the rate of clot formation, and the bottom panel shows the maximum clot strength (G_max_); alpha and G_max _are shown over a titrated range of rThrombin activity that was added to the TEG reaction.

Increasing amounts of titrated rThrombin activity led to increased rates of clot formation (measured by the alpha angle; Figure 4, top panel; r = 0.72; p = 0.002) and clot strength (shear elastic modulus, defined as G_max_; Figure 4, bottom panel; r = 0.77; p < 0.001) in heparin + clopidogrel blood samples. In these in vitro clotting studies, sequential increases in the amount of rThrombin activity ultimately led to maximum (plateau) values for the rate of clot formation and clot strength.

## Discussion

Numerous factors work to reduce both endogenous and exogenous thrombin concentration at the bleeding wound interface, including removal and dilution by hemorrhagic blood flow, rapid binding to inhibitors such as antithrombin III, entrapment in the developing thrombus, or mechanical removal by sponge and/or irrigation. Whether topical thrombin is applied or not, surgeons rely on intraoperative gross evaluations of hemostasis as predictors of whether hemostasis will be durable after wound closure. When hemostasis is delayed, laboratory assessments may be performed to rapidly detect any pathologic derangements in clotting. However, the results do not always correlate with intraoperative bleeding severity, nor do they reliably predict response to surgical intervention because of the many variables influencing clot formation at the wound. We addressed several of these variables in this study by examining the effects of rThrombin concentration on hemostasis in animals treated with commonly-administered anticoagulation agents.

Increasing concentrations of rThrombin decreased TTH in a concentration-dependent manner in a rabbit model of arterial bleeding. Treatment of heparinized animals with clopidogrel delayed the onset of hemostasis. However, treatment with rThrombin at a concentration of 1000 IU/mL mitigated the delay in onset of hemostasis, consistently providing comparable results whether animals were treated with heparin or with heparin + clopidogrel. This suggests that the efficacy of 1000 IU/mL rThrombin as a topical hemostatic agent was unaffected by clopidogrel-induced platelet inhibition. In contrast, the application of 125 IU/mL rThrombin resulted in a more variable and delayed hemostatic response, suggesting that this concentration of rThrombin was too low to reliably overcome clopidogrel-induced platelet inhibition. This is the first report of thrombin concentration-dependent mitigation of the delayed hemostasis resulting from platelet inhibition. This observation lends credence to the widespread use of topical thrombin preparations in vascular and cardiac surgery, where platelet dysfunction is common, and supports the proposition that use of thrombin at 1000 IU/mL evolved because of reliable efficacy over a wide range of clinical settings, including both pathologic and pharmacologically-induced clotting abnormalities.

The broad utility of the 1000 IU/mL concentration of rThrombin was further supported by an evaluation of clot durability that was performed by clamping the graft following the achievement of hemostasis (clot burst testing). Clot burst is thought to reflect the adhesiveness of the clot boundary to the PTFE graft material, the resistance of the clot to mechanical disruption, and indirectly, the development of platelet force. Development of platelet force is a process of thrombus maturation in which platelet contraction leads to increased fibrin strand density, thus ensuring that a sudden increase in blood pressure does not lead to clot failure. Normal platelet function is required for physiologic clot initiation [18]. Furthermore, pharmacological or mechanical inhibition of platelet function has been associated with increased bleeding in humans after cardiopulmonary bypass [19]. Persistent hemostasis that resists the stresses of the early recovery period is a desirable outcome for all surgical procedures. We observed that clots formed in the presence of 1000 IU/mL rThrombin were significantly more resistant to disruption than were clots formed in the presence of 125 IU/mL rThrombin in animals treated with heparin + clopidogrel. The disruption of clots formed in the presence of 125 IU/mL rThrombin was indistinguishable from the disruption of clots formed in the presence of placebo. These findings are consistent with those of other investigators; differences in clot structure and maturation at varying thrombin concentrations have been well described [6-8]. The fragile state of hemostasis that was achieved with 125 IU/mL human thrombin and AGS by Adams, et al. can be better understood in light of our observations [11]. The rebleeding wounds observed at 12 minutes in the Adams porcine hepatic bleeding model may have rebled secondary to inadequate clot strength and density and a higher concentration of thrombin might have resulted in more persistent hemostasis.

Finally, results from the in vitro TEG technique are consistent with prior reports that fibrin clot strength is a function of available thrombin concentration, suggesting that there is an optimal concentration range for rThrombin efficacy [6-8]. Although the quantities of rThrombin used to initiate clotting in the TEG experiments cannot be directly compared with that used for hemostasis in the rabbit AV shunt model, increased rates of clot formation and improved clot strength in the TEG experiments was achieved with increasing amounts of rThrombin activity added at the time of clot initiation. These results are also consistent with those reported for human blood samples in which TEG parameters increased across a 10-fold range of rThrombin concentrations [20].

## Conclusion

In an animal model of vascular bleeding, rThrombin at a concentration of 1000 IU/mL reliably shortened TTH and resulted in more durable hemostasis in the presence of heparin anticoagulation and clopidogrel platelet inhibition when compared with rThrombin at a concentration of 125 IU/mL. This observation has significant implications for current clinical practice. Increasing numbers of patients are presenting for emergent, urgent, or elective surgical procedures with significant clopidogrel platelet inhibition as a result of a reluctance or inability to withhold clopidogrel prior to surgery. Results in this rabbit AV shunt model suggest that topical rThrombin at a concentration of 1000 IU/mL may reliably provide hemostasis in surgical patients who have received clopidogrel, a finding that should prompt further clinical investigation.

## Competing interests

All authors were employees and stockholders of ZymoGenetics, Inc. at the time of this research.

## Authors' contributions

SDH and WAA participated in the design and coordination of the study and statistical analysis. PDB designed the in vitro TEG assays. RG designed and performed the in vivo rabbit assays and clot burst experiments. TZ designed and performed the statistical analyses. All authors were active in drafting the manuscript, and all authors read and approved the final manuscript.
